# Binding Revisited—Avidity in Cellular Function and Signaling

**DOI:** 10.3389/fmolb.2020.615565

**Published:** 2021-01-14

**Authors:** Simon Erlendsson, Kaare Teilum

**Affiliations:** ^1^Structural Studies Division, Medical Research Council Laboratory of Molecular Biology, Cambridge, United Kingdom; ^2^Structural Molecular Biology Group, Novo Nordisk Foundation Centre for Protein Research, Faculty of Health and Medical Sciences University of Copenhagen, Copenhagen, Denmark; ^3^Structural Biology and NMR Laboratory and the Linderstrøm-Lang Centre for Protein Science, Department of Biology, University of Copenhagen, Copenhagen, Denmark

**Keywords:** avidity, functional affinity, retention time, cellular avidity, modeling avidity

## Abstract

When characterizing biomolecular interactions, avidity, is an umbrella term used to describe the accumulated strength of multiple specific and unspecific interactions between two or more interaction partners. In contrast to the affinity, which is often sufficient to describe monovalent interactions in solution and where the binding strength can be accurately determined by considering only the relationship between the microscopic association and dissociation rates, the avidity is a phenomenological macroscopic parameter linked to several microscopic events. Avidity also covers potential effects of reduced dimensionality and/or hindered diffusion observed at or near surfaces e.g., at the cell membrane. Avidity is often used to describe the discrepancy or the “extra on top” when cellular interactions display binding that are several orders of magnitude stronger than those estimated *in vitro*. Here we review the principles and theoretical frameworks governing avidity in biological systems and the methods for predicting and simulating avidity. While the avidity and effects thereof are well-understood for extracellular biomolecular interactions, we present here examples of, and discuss how, avidity and the underlying kinetics influences intracellular signaling processes.

## Introduction

Cell function relies on meticulously timed dynamic networks of biomolecular interactions taking place in changeable cellular compartments with great variability in size, shape, pH, solute concentration, and molecular crowding. All these parameters affect diffusion and reaction rates. For instance, biomolecular interactions where one or more of the involved species are linked to membranes will display very different binding strength and kinetics compared to interactions occurring in solution within the cytosol. Here we use the term reaction to describe processes where intermolecular interactions are formed or broken.

**Figure 1:**
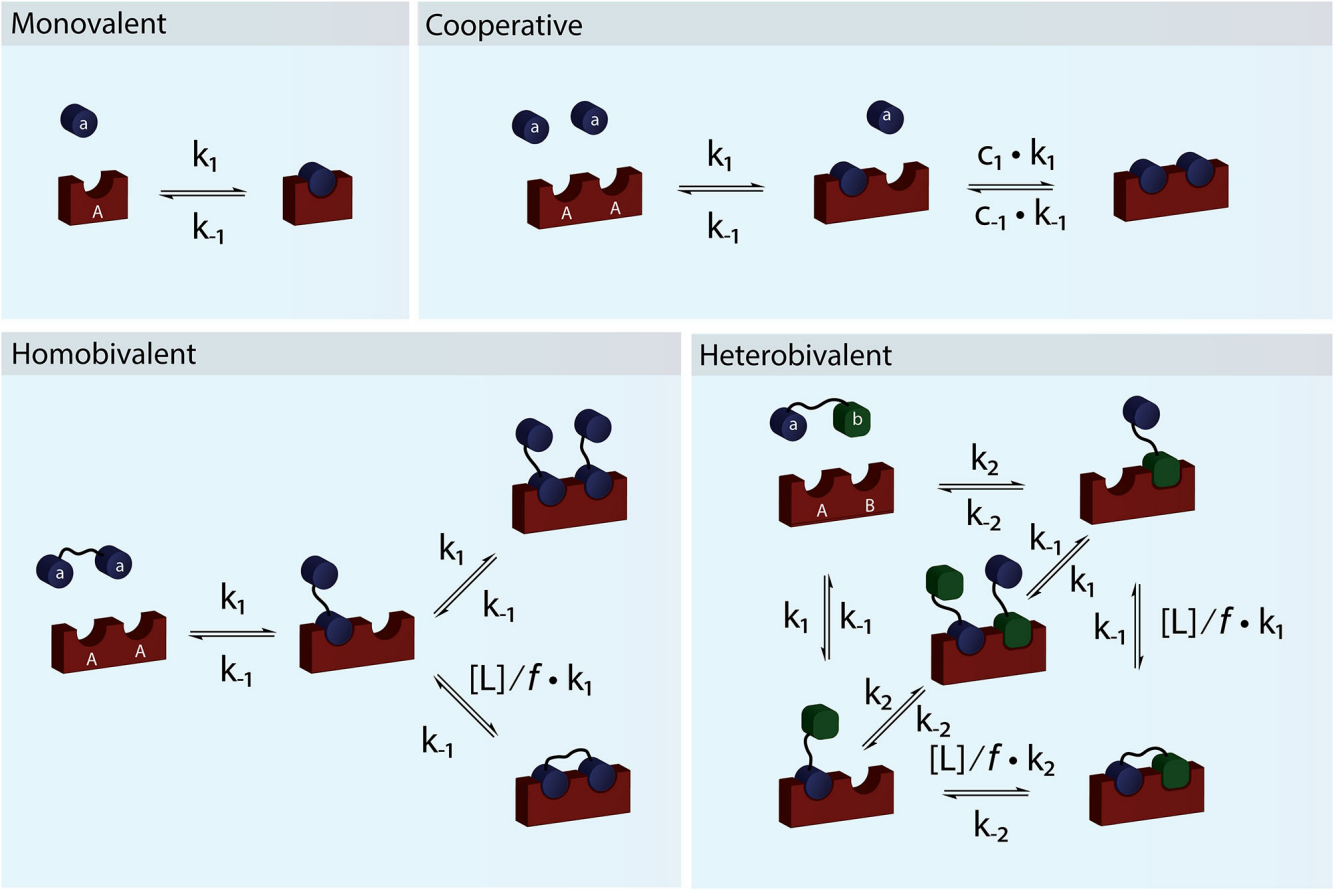
Schematic figure showing monovalent, cooperative and homo- and heterobivalent reactions. In cooperative binding either one or both the association and dissociation rates of the second binding step may be modulated by cooperativity factors c_1_ and c_−1_. Only cases where only one of the interacting species are multitopic and where the reaction is not diffusion limited allows for accurate determination of the cooperativity. In multivalent binding, both of the interacting species are multitopic. For simplicity we have presented reaction schemes for both a homo- and heterobivalent interactions. In these cases the association rate of the second binding step is modulated by the local concentration, [L], and an empirical penalty factor, *f*. Multivalent interactions can also be cooperative but the direct effect is difficult to determine accurately.

**Figure 2:**
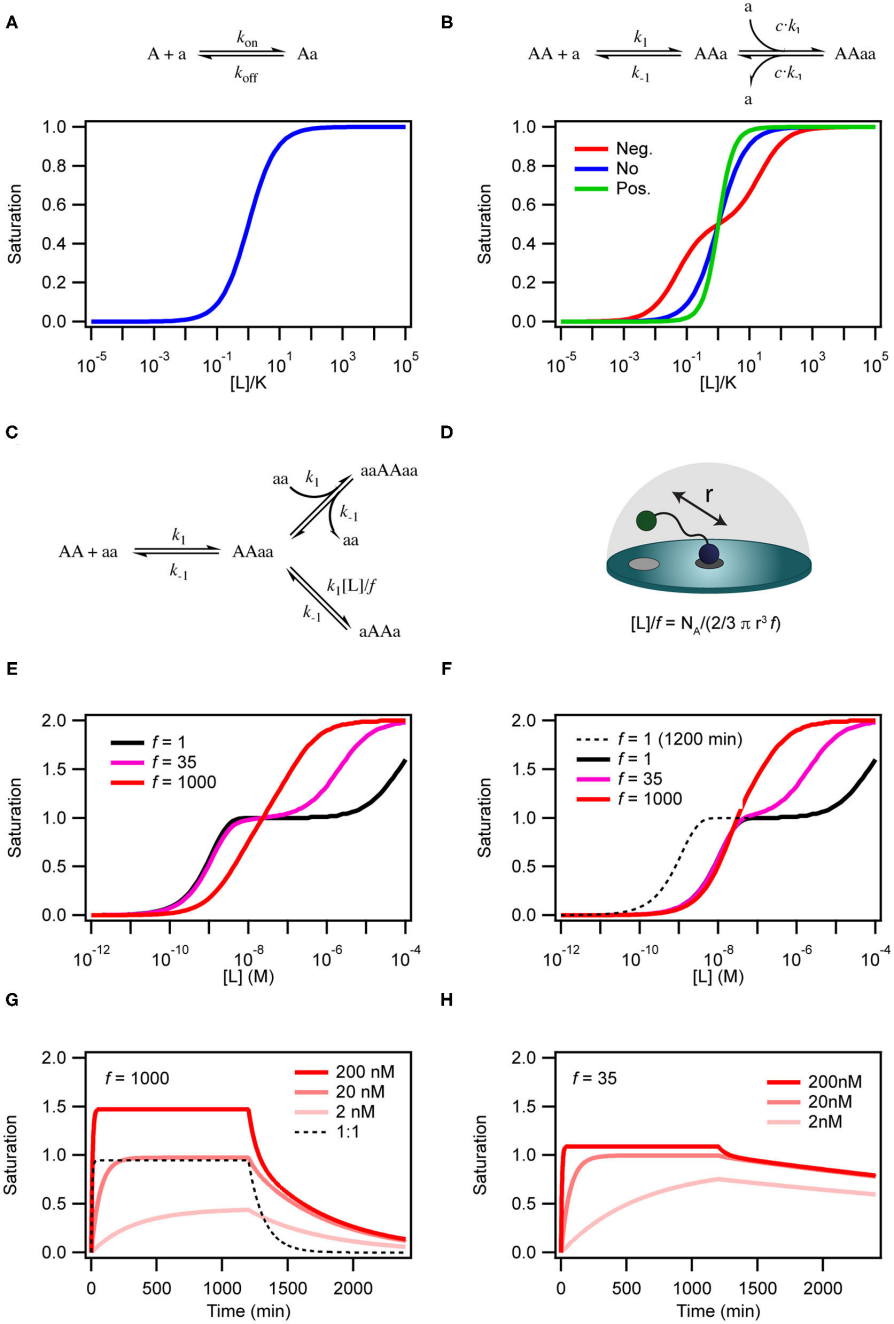
Simulations of binding curves. **(A)** Equilibrium binding curve for a monovalent ligand binding to a monovalent receptor. **(B)** Equilibrium binding curves for a monovalent ligand binding to a dimeric (or divalent) receptor in case of negative cooperativity, *K*_d1_ = 0.01*K*_d2_ (red), no cooperativity (blue) and positivity cooperativity, *K*_d1_ = 100*K*_d2_ (green). In the case of cooperativity, the scale on the x-axis is calculated with *K* = √*K*_d1_*K*_d2_. For the non-cooperative case *K* = *K*_d_. **(C)** Model for binding of a homo-divalent ligand to a homo-divalent receptor. All steps are assumed to have the same microscopic rate constants, *k*_1_ and *k*_−1_. The intra-molecular binding reaction AAaa to aAAa is also controlled by the effective ligand concentration [L] and the empiric proximity factor *f* that accounts for steric restrictions on the intramolecular process compared with the intermolecular process. **(D)** For the intramolecular binding to a receptor on a surface, the local concentration is calculated as the concentration of the ligand in half-sphere with a radius equal to the distance between two binding sites on the ligand when it is fully extended. **(E)** Binding curves for the model in **c** with [AA] = 0.1 nM *k*_1_ = 1.85 × 10^5^ M^−1^min^−1^ and *k*_−1_ = 8.5 × 10^−3^ min^−1^ at three different values of *f* and [L] = 136 μM simulated after 1,200 min equilibration. **(F)** Same as in **(E)** except that the curves were extracted after 120 min. **(G)** Time traces of binding and dissociation for the model in **(C)** at three different ligand concentrations, with *f* = 1,000 and other parameters as in **(E)**. For comparison the time trace for at 1 to 1 binding reactions with *k*_on_ = 1.85 × 10^5^ M^−1^min^−1^ and *k*_off_ = 8.5 × 10^−3^ min^−1^. **(H)** Same as in **(G)**, except *f* = 35. ll simulations were performed with COPASI (Hoops et al., 2006).

The strength of a biomolecular interaction is commonly referred to as the affinity. The affinity is quantified as the equilibrium constant, *K*_a_, for the reaction *A* + *a* ⇌ *Aa* (Figures 1, 2A). Often the equilibrium constant for the reverse reaction, the dissociation constant, *K*_d_, is used instead. The microscopic rate constants for the above reaction are called the association rate constant, *k*_on_, and dissociation rate constant, *k*_off_. The relation between these parameters and the equilibrium concentrations of species A, a and Aa are given by:

(1) $$\begin{array}{l} K_{d}=\frac{1}{K_{a}}=\frac{k_{off}}{k_{on}}=\frac{\left[A\right]\left[a\right]}{\left[Aa\right]} \end{array}$$

The association and dissociation rates depend on the type of interaction and external physical properties (Calef and Deutch, 1983) and may span several orders of magnitude. *k*_on_ ranges from 10^9^ M^−1^s^−1^ for the fastest diffusion limited reactions that are enhanced by electrostatic interactions to 10^4^ M^−1^s^−1^ for slow reactions that often are governed by large conformational changes (Schreiber and Fersht, 1996; Dogan et al., 2014). *k*_off_ depends on how fast the intermolecular interactions that stabilize the complex are broken and typically ranges between 10^4^ and 10^−4^ s^−1^. Similar to the large variation in the underlying rate constants, the *K*_d_ vary over several orders of magnitudes from around 1 mM typically for protein-carbohydrate interactions to around 1 fM for the binding of some metal ions. Consequently, *K*_d_ values may be the result of very different microscopic rate constants.

Determining affinities and underlying rate constants often require the interacting molecules to be recombinantly expressed, purified and titrations to be performed in an isolated and artificial environment. In most cases, this approach provides affinities and rate constants identical or comparable to the when the interactions happen within or on a real cell. In other cases, cooperativity, valency, and the local environment will result in the “real” functional affinities being radically different from those obtained by *in vitro* methods. The terms *Functional affinity* and *Avidity* have been used interchangeably to describe how the affinity changes when the molecules are observed in their native environment. In particular, the intrinsic affinity and the avidity will be radically different in multivalent systems where both molecules have two or more linked binding sites. In such cases low affinity (μM-mM) of the individual binding events can translate into very a high (pM-nM) avidity for the multivalent system. The local cellular compartment where the interaction takes place can also greatly affect the avidity. In the following, we will focus on how cooperativity, multivalency and diffusional properties affect the avidity and the underlying microscopic rate constants and review various routes for predicting and modeling avidity. Finally, we present examples on how avidity has been found to modulate intracellular interactions important for maintaining proper cellular function and signaling.

## Multivalency and Cooperativity—How They are Different But Not Mutually Exclusive

For a system to be cooperative, at least one of the interacting partners e.g., a receptor must have multiple interaction sites (be multitopic), which can be occupied by monomeric species (Figure 1). In a cooperative system, binding of one ligand must alter the affinity for the following binding event (Hunter and Anderson, 2009). The most classical example of (positive) cooperativity is hemoglobin, where binding of oxygen by one of the four subunits promotes a conformational change, which increases the affinity for oxygen of the adjacent subunit (Lee and Karplus, 1983).

The simplest situation where cooperativity can be observed is when a dimeric protein binds two ligands for which the overall process may be described by the reaction: *AA* + 2*a* ⇌ *AAaa*. In this case, the reaction scheme is slightly misleading as it suggests that both ligands bind simultaneously. Even with a diffusion limited on-rate the likelihood of two ligand molecules binding at the same time is extremely low and the process will therefore be very slow and practically never reach equilibrium. Sequential binding of the two ligands is much more likely in a process described by the reaction scheme shown in Figure 2B. If the two binding events are independent and described by the same rate constants, the process is completely un-cooperative and the binding curve will be similar to the binding curve for the simple 1:1 binding above (Figure 2B, blue curve). If on the other hand binding at one site leads to *allosteric* structural changes in the other binding site that results in changed rate constants of the second binding event, the system is *cooperative*. The cooperativity can affect both the association and dissociation rates and be either positive (higher affinity) or negative (lower affinity).

For a system to be multivalent, both the receptor and the ligand must be multitopic (Figure 1). Multitopic species can be either covalently attached (In a multitopic protein or as distinct DNA binding motifs) or tethered (e.g., in a membrane) and for this reason, the microscopic rate constants can be the result of many different system or environmental specific parameters i.e., Covalently attached multitopic binders may experience very different degrees of flexibility and intermolecular dynamics and membrane tethered ligands diffuse more slowly and only in two dimensions compared to molecules in solution. Multivalent systems can be either homovalent (identical binding sites) or heterovalent (different binding sites) and also appear as positive and negative cooperative (Figure 1). Thus, a homobivalent system can have different rate constants for the first and second binding event (Vauquelin et al., 2014). Cooperativity, however, can be very hard to show in multivalent systems. The simplest situation where multivalency can be observed is when a homo-dimeric protein (**AA**) binds a homo-dimeric ligand (**aa**) according to the scheme presented in Figures 1 and 2C. As the heterogeneity or valency increases, the number of possible bound states increases exponentially. Not considering any cross binding (several receptors bound by the same ligand), the number of possible states can be estimated by the expression $n_{states}\left(n\right)=1-n+n2^{n}$. For a heterobivalent interaction, the number of possible states is thus 7, which increases to 156 for a pentavalent interaction (Erlekam et al., 2019). The initial binding step of any multivalent interaction is governed by the intrinsic association rate constant. After binding of the first ligand, the second ligand is spatially restricted and in close proximity to its target. The effect of this forced proximity can be formalized into a local concentration, [L], and therefore the effective association rate of the second ligand binding will proceed much faster than the first (Kramer and Karpen, 1998). In the case of dissociation of only one ligand from its target, which is independent on the local concentration and governed the intrinsic dissociation rate, k_off_, the ligand will still maintain the high local concentration and the probability of rebinding to the same or other sites in the proximity of the multivalent molecule will be high. For this reason, multivalent interactions can have extremely high residence times and may be virtually irreversible (Vauquelin and Charlton, 2013; Huskens, 2018). In almost all cases, the local concentration will be much higher than the bulk ligand concentration (typically around μM) and therefore the rebinding event will be favored over the association of a third multivalent molecule (ternary complex). In such cases, the intermediate product **aaAA** will be short-lived and the system will quickly equilibrate and form the closed binary complex where each binding site on **aa** interacts with a binding site on **AA**. We call this complex **aAAa**. If, however, the local concentration of free **aa** is very high or the formation of the multivalent complex is very unfavorable (*f* is high), the ternary complex, **aaAAaa**, will be populated. Such a multivalent system will take much longer to equilibrate and consequently, biological multivalent systems may never fully reach equilibrium (Vauquelin and Charlton, 2013). The occupancy and life-times of the intermediate **aaAA** and ternary **aaAAaa** products, can be of great biological significance (Erlendsson et al., 2019).

## Hindered Diffusion and Reduced Dimensionality

In a real biological system knowing the free receptor and ligand concentrations is not straightforward. Cells and tissue are highly compartmentalized, and fluctuations in temperature, pressure, ionic concentrations, pH and oxidation levels as well as the dimensionality and molecular composition of the compartment are crucial regulators of cellular function. All of these parameters govern the diffusional properties and flux of molecules and therefore also the avidity of both mono and multivalent interactions through modulation of the effective association rate constants (Goldstein and Dembo, 1995). For example, interactions taking place in confined protein dense areas beneath or above the plasma membrane (immunological synapses, synaptic clefts or the postsynaptic density) will often suffer from reduced dimensionality and hindered diffusion which can result in a concentration gradient between membrane proximal and distal regions (Vauquelin and Charlton, 2010). *Coombs and Goldstein* (Goldstein and Dembo, 1995; Coombs and Goldstein, 2004) proposed that the effect of hindered diffusion can be approximated by calculating modulated rate constants considering the dimensions of the compartment, the flux and the diffusion coefficients:

(2) $$\begin{array}{l} k_{on}^{diff}=\frac{k_{on}}{1+\frac{\left[A\right]k_{on}}{k_{+}}} \end{array}$$

(3) $$\begin{array}{l} k_{off}^{diff}=\frac{k_{off}}{1+\frac{\left[A\right]k_{on}}{{\bar{k}}_{+}}} \end{array}$$

Where *k*_+_ is the average diffusion-limited association constant within the compartment and ${\bar{k}}_{+}\;$ is the average diffusion-limited rate constant for leaving the compartment. Using these modified rate constants in simulations Vauquelin et al. showed that hindered diffusion can increase the time before equilibrium is reached and prolong the residence time of the ligands close to their targets much like multivalent rebinding (Vauquelin and Charlton, 2013). On the other hand, the avidity can be decreased by hindered diffusion when the ligand concentration and flux is lower than the association rate. Consequently, hindered diffusion and reduced dimensionality can indirectly either enhance or decrease the avidity, even in monovalent cases. The concentrations of receptors and ligand and their diffusional properties are compartment specific and therefore the avidity can be hard to predict at e.g., neuronal synapses or immunological synapses (Caré and Soula, 2011; Eloul and Compton, 2016; Eloul et al., 2016; Li et al., 2018).

## Measuring and Modeling Avidity

In practice it is not straight forward to get a clean readout of the avidity of multivalent systems. When the multitopic species are covalently attached and not tethered in the plasma membrane, the underlying kinetics of multivalent interactions can typically be assessed by in-solution fluorescence methodologies, such as fluorescence anisotropy, stopped flow fluorescence, FRET and fluorescence correlation spectroscopy. Obtaining the microscopic rate constants requires purification of one or more of the species and also require systematically removing or mutating each individual binding site to get the microscopic rate constants.

When one of the multitopic species are not covalently attached, surface plasmon resonance (SPR) (Lynch et al., 2014; Akkilic et al., 2019; Porkolab et al., 2020) is currently most used. SPR is label-free and allows for immobilization of the monovalent species (the receptor) at a given density on an activated surface. Following immobilization, the multitopic ligand can then be titrated over the receptor surface. By varying both the density of the receptor, and the concentration and flow of the ligand this can provide both the intrinsic affinity and avidity of the interaction. Another related label-free method is the Quartz crystal microbalance (QCM-D) (Decker et al., 2003; Johansson, 2010) which can be utilized for obtaining affinities over a wide range of biomolecular interactions. Both systems can deal with purified receptors reconstituted in membrane mimetics and also allows for formation of lipid bilayers (Cho et al., 2010; Patching, 2014; Bocquet et al., 2015; Brun et al., 2015; Parkkila et al., 2018; Iorio and Huskens, 2020). Other more recent methods for measuring avidity use confocal or TIRF microscopy to obtain avidities in the native cellular environment (Oh et al., 2012; Erlendsson et al., 2019). While these systems are more heterogenous and the results harder to interpret, they maintain the complexity and integrity of a native cellular system, and also allow for probing ligand binding to both intra and extracellular membrane tethered multivalent species. Finally, atomic force microscopy (AFM) has also been utilized to obtain avidities of antibody-antigen interactions or cell-cell adhesion strength (Wojcikiewicz and Moy, 2005; Zhang et al., 2020).

The avidity is a complex entity governed by the valency, cooperativity and the local compartment. Therefore, to model the avidity of a receptor-ligand interaction several factors must be considered: (1) The intrinsic affinity of the involved individual complex(es), (2) the valency of the interaction partners, (3) structural re-arrangements of proteins (conformational exchange), and (4) diffusional properties and reduced dimensionality. These contributions are not easily deconvoluted, but a detailed experimental analysis combined with a thermodynamic or kinetic model can identify rate limiting steps and the sensitivity of the system to changes in intrinsic affinity. Using thermodynamics to describe avidity is particularly useful when the valency, *n*, is high (> 5). Thermodynamics, however, only give steady-state equilibrium information. Kinetic models have the advantage that also non-equilibrium situations can be described. A drawback of using reaction kinetic models, is that for systems with high valency the differential equations (see Equations 7–11) become very complex and computationally challenging to analyse (Vauquelin and Charlton, 2013). Below we will describe how thermodynamic models and reaction kinetic models can be used to predict and model the avidity.

### The Thermodynamic Model

For a multivalent receptor-ligand (denoted R and L, respectively) interaction process, like for any other process at constant pressure, the Gibbs free energy consists of an enthalpic and an entropic contribution connected by the fundamental relation Δ*G*^0^ = Δ*H*^0^-*T*Δ*S*^0^. Considering the contributions to each of these terms will provide some insight into what determines the avidity of a multivalent reaction. First, it is important to clearly define the reaction for which Δ*G*^0^ is calculated. An *n*-valent binding reaction may be written as:

(4) $$\begin{array}{l} \text{R}+\text{L}=\left[\text{RL}\left(1\right)+\text{RL}\left(2\right)+\ldots \text{RL}\left(n-1\right)+\text{RL}\left(n\right)\right] \end{array}$$

where the number in parenthesis is the number of ligand sites bound to the receptor. For this reaction, the total change in free energy, Δ$G_{\text{total},}^{0}$ for the process could be defined as the difference in energy between the free species and the fully bound state where all *n* binding sites are occupied corresponding to the process R + L = RL(*n*). Δ$G_{\text{total}}^{0}$ is then a result of all the individual contributions from the *n* binding sites. In some instances, the avidity is defined as the equilibrium constant for this process, i.e. *K*_av_ = exp(−Δ$G_{\text{total}}^{0}$/RT) (Mammen and Choi, 1998; Krishnamurthy et al., 2006), which makes sense if the biological effect depends on complete binding of all *n* binding sites.

However, if *n* is large, the intermediate states will dominate, and the fully bound state will only be sparsely populated. In addition, many binding assays measure the total concentration of bound receptors (i.e., the sum of all bound states) and the concentration of free receptor. In these cases, the avidity constant, *K*_av_, can be defined as the equilibrium constant for the equilibrium between free and any bound receptor species (Kitov and Bundle, 2003; Sriram et al., 2009; Kane, 2010).

(5) $$\begin{array}{l} K_{av}=\frac{\left[R_{bound}\right]}{\left[R_{free}\right]\left[L\right]}=\frac{\sum_{i=1}^{n}\left[RL_{i}\right]}{\left[R_{free}\right]\left[L\right]} \end{array}$$

where *i* is the number of occupied sites on the receptor. For each of the bound states the binding energy can be expressed as a function of *i* (Krishnamurthy et al., 2006):

(6) $$\begin{array}{l} \Delta G_{multi}^{0}\left(i\right)=-RT\ln\frac{\left[RL_{i}\right]}{\left[R_{free}\right]\left[L\right]}\\ \;=i\Delta G_{affinity}^{0}+\left(i-1\right)[T\Delta S_{trans/rot}^{0}+\Delta H_{linker}^{0}\\ \;-T\Delta S_{linker}^{0}+\Delta G_{coop}^{0}]-RTln\left(\Omega_{i}\right) \end{array}$$

In the last part of this equation the energy is broken down to six individual terms. The value of the individual terms can be difficult to determine, particularly for biological ligands where compounds with different and well-defined valency cannot readily be produced. Still, discussing how each term contributes to $\Delta G_{multi}^{0}$ gives an understanding of the molecular mechanisms underlying the magnitude of the avidity effect. The first term in equation 6 is simply the binding energy for the receptor interacting with *i* monomeric ligands that each has a single binding site. When the ligand is also multitopic, only the first interaction thermodynamically resembles the interaction of a monomer. For the remaining *i* – 1 interactions the binding energies are modified by the terms in squared brackets. The first of these terms is the rotational and translational entropy. This entropy is only lost in the first interaction and therefore should be subtracted –(–TΔ*S*^0^) from the remaining *i* – 1 interactions. The second term is the enthalpic contribution from interactions between the linker parts of the ligand and the receptor that could be either favorable or unfavorable. To connect *i* binding sites, *i* – 1 linkers are needed and therefore this is added (*i* – 1) times. The third term is the conformational entropy of the linkers, which often will only be marginally affected in the first binding event. For the *i* – 1 subsequent binding events, the change will be much larger as the binding sites become fixed relative to each other. Typically, the Δ$H_{\text{linker}}^{0}$ and Δ$S_{\text{linker}}^{0}$ terms will be highly correlated through entropy-enthalpy compensation (Williams et al., 2004; Teilum et al., 2009). However, depending on specific conformational changes occurring in the linker in each binding step, the specific enthalpic and entropic contributions are hard to predict (Teilum et al., 2009). The last term in the brackets is the effect of cooperativity, which is normally small relative to the other terms (Krishnamurthy et al., 2006; Fasting et al., 2012). The very last term relates to the degeneracy, Ω_I_, of the system, which is how many different ways the *i* interactions between the receptor and the ligand can be formed. The degeneracy can be considered an additional entropy term and is called the avidity entropy in the literature (Kitov and Bundle, 2003; Martinez-Veracoechea and Leunissen, 2013). In detailed simulations of avidity, it is often useful to apply statistical thermodynamics. Please see (Xu and Shaw, 2016; Curk et al., 2018) for examples hereof.

A much simpler but less informative evaluation of the avidity in a multivalent system, which is however practically tractable, is to calculate the enhancement of the affinity relative a monovalent receptor-ligand interaction as β = *K*_av_/*K*_mono_. Enhancements in the range β = 10^3^-10^17^ have been observed for even simple di- and tri-valent systems (Rao et al., 2000; Erlendsson et al., 2019).

### The Reaction Kinetic Model

Whereas, the thermodynamic description of a receptor-ligand interaction provides insight into the forces that drive the reaction, it does not tell about the order of the events. For this a kinetic model is necessary. Simulations of the kinetic models rely on sets of differential equations representing each state as a function of time (see below). We used kinetic simulations to obtain binding curves shown in Figure 2. All equations represents one-step, reversible interactions obeying the laws of mass-action. By consecutively solving the differential equations in parallel, all states can be monitored over time (Hoops et al., 2006; Vauquelin and Charlton, 2010, 2013). As kinetic models easily become very complex and difficult to interpret we here consider the interaction of a homodimeric receptor (**AA**) with a dimeric ligand (**aa**). We assume that there is no allostery and that both sites on the receptor and the ligand are identical. The reaction scheme is shown in Figure 2C and the differential equations describing the species are:

(7) $$\begin{array}{l} \frac{d\left[AA\right]}{d\left(t\right)}=k_{-1}\left[aaAA\right]+k_{-1}\left[AAaa\right]-{2\;k}_{1}\left[aa\right]\left[AA\right] \end{array}$$

(8) $$\begin{array}{l} \frac{d\left[aaAA\right]}{d\left(t\right)}=k_{1}\left[aa\right]\left[AA\right]+k_{-1}\left(\left[aAAa\right]+\left[aaAAaa\right]\right)\\ \;-k_{-1}\left[aaAA\right]-\left(\frac{\left[L\right]k_{1}}{f}\right)\left[aaAA\right]\\ \;-k_{1}\left[aaAA\right]\left[aa\right] \end{array}$$

(9) $$\begin{array}{l} \frac{d\left[AAaa\right]}{d\left(t\right)}=k_{1}\left[aa\right]\left[AA\right]+k_{-1}\left(\left[aAAa\right]+\left[aaAAaa\right]\right)\\ \;-k_{-1}\left[AAaa\right]-\left(\frac{\left[L\right]k_{1}}{f}\right)\left[AAaa\right]\\ \;-k_{1}\left[AAaa\right]\left[aa\right] \end{array}$$

(10) $$\begin{array}{l} \frac{d\left[aAAa\right]}{d\left(t\right)}=\left(\frac{\left[L\right]k_{1}}{f}\right)\left[aaAA\right]\\ \;+\left(\frac{\left[L\right]k_{1}}{f}\right)\left[AAaa\right]-2k_{-1}\left[aAAa\right] \end{array}$$

(11) $$\begin{array}{l} \frac{d\left[aaAAaa\right]}{d\left(t\right)}=k_{1}\left[aAA\right]\left[aa\right]+k_{1}\left[AAaa\right]\left[aa\right]\\ \;{-2k}_{-1}\left[aaAAaa\right] \end{array}$$

There are two steps that depend on the concentration of free ligand; (i) the initial interaction between **AA** and **aa** to form the binary **AAaa** (or **aaAA**) complex and (ii) the binding of a second ligand to form the ternary complex **aaAAaa**. This **aaAAaa** state will form when the concentration of the free ligand **aa** is high in solution. At low concentrations of **aa**, intra-complex formation of an interaction between the second subunit on the protein and the second subunit on the ligand (**aAAa**) may be more favorable than formation of the ternary complex. The formation of this species is dependent on the effective local concentration experienced by the second binding site on the protein relative to the concentration of the ligand free in solution. In the model in Figure 2C the effective local concentration is expressed as [L]/*f*. For a ligand bound to a receptor at a membrane surface, [L], is the concentration of the second binding site of the ligand in a half-sphere centered at the occupied binding site of the ligand and with a radius, r, equal to the distance between the two binding sites on the ligand (Figure 2D). *f* is an empirical penalty factor that accounts for geometrical constraints on where in the half sphere the free ligand site may actually be found. If the local concentration of the second binding site is very high compared to the free concentration of ligand and also large compared to *k*_1_/*k*_−1_ the closed dimeric complex (**aAAa**) will dominate. In a titration experiment, this situation corresponds to the black line in Figure 2E where *f* =1, which also results in a very high avidity. The ternary complex will only become populated at extreme ligand concentrations and the binding curve resembles that of a system in negative cooperativity. If on the other hand the effective local concentration of the second ligand binding site is very small, the closed dimeric complex will not be populated before the concentration of free ligand favors formation of the ternary complex (Figure 2E, red line). For the less strong local concentrations (high r of *f*) both the closed dimeric complex and the ternary complex are populated, and the dominating species changes over a relative narrow concentration range, but still with a two-step behavior similar to negative cooperativity (Figure 2E, magenta line).

The kinetics of the binding between a dimeric protein and a dimeric receptor (Figure 2G, red curves) is clearly different from the kinetics of a single step binding between two monomers (Figure 2G, black dashed curve). It is particularly clear that the dissociation kinetics in addition to being biphasic also becomes very slow as a consequence of rebinding of the partly dissociated ligand. If the closed dimeric complex is favored (by lowering *f* to 35 as in Figure 2H) the dissociation is extremely slow and will take several days to complete (Radnai et al., 2010; Li et al., 2014; Xu and Shaw, 2016; Erlendsson et al., 2019; Errington et al., 2019; Sørensen and Kjaergaard, 2019).

Moreover, the time before the system reaches equilibrium increases for the dimeric binding models when *f* is decreased. This is particularly pronounced at low ligand concentrations. Indeed, for a binding reaction following the model in Figure 2C, the outcome of a titration experiment is very different if samples are equilibrated for 2 h (Figure 2E) or 20 h (Figure 2F). Thus, many biological multivalent interactions will effectively be non-equilibrium binding events. With a slow macroscopic association rate, it may in some cases take several hours or even days to reach equilibrium (Vauquelin and Charlton, 2013).

## Avidity in Biology

Avidity was initially conceptualized to explain how neutralizing bivalent antibodies increase efficacy *in vivo* when compared to the affinity experimentally determined *in vitro* (Finkelstein and Uhr, 1966; Hornick and Karuch, 1972; Karush and Hornick, 1973), and the theoretical frameworks are well adapted to model antibody avidity and for designing potent multivalent antibodies (Cuesta et al., 2010; Baran et al., 2017; Einav et al., 2019). Antibodies have low valences (up to 10 for IgM) and the interactions themselves are highly dependent on the abundance and spatial organization of their Fc receptor targets as the two binding sites of a typical bivalent IgG antibody are no more than 10 nm apart. Having a short distance between the binding sites results in very high local concentrations and thus the rebinding probability governing the second binding event becomes very high.

Other well-studied multivalent systems are cell-cell, cell-bacteria and virus-host interactions (Westerlund and Korhonen, 1993; Choi et al., 1996; Kitov et al., 2000; Zhang et al., 2002; Sieben et al., 2012; Xiong et al., 2013; Einav et al., 2019). The avidity of these systems depends largely on the abundance and organization of the relevant receptors on the cell surfaces (Xu and Shaw, 2016). Cells and bacteria display thousands of receptors facilitating a wealth of homo- and heteromultivalent interactions. The intrinsic affinities of these systems are often low (mM) but can be higher depending on the sparsity of the receptors. Often 10–1,000 receptors are involved in forming tight connections between cells and it is not unusual to have avidities 1–10 million-fold higher than the intrinsic affinity of the monovalent ligand (β of 10^6^-10^7^). Some of these ultrahigh avidity interactions display high degrees of degeneracy and are virtually not reversible processes. They are only broken by shear force, changes in local environment (pH, ionic strength), or enzymatic cleavage.

Less degenerate complexes rely on strict spatial coordination of multiple interaction pairs. In such cases even subtle changes in intrinsic affinities or geometry may change binding preference. This can be the case for some virus-host interactions where the mutational rate is high, and the density of glycoproteins and receptors can vary significantly across different viral strains and cell types. As an example, it was shown that for avian influenza H5N1 even small changes in haemagglutinin-SA binding affinity is sufficient to switch the binding preference of the virus from avian receptors to human receptors (Xiong et al., 2013).

The above types of multivalent systems have been thoroughly reviewed in several excellent papers (Mammen and Choi, 1998; Kitov and Bundle, 2003; Fasting et al., 2012) and while they are indeed very diverse in terms of number of molecules, specificity and affinity, they are all extracellular.

## Intracellular Avidity

Many intracellular proteins are composed by multiple domains (Sriram et al., 2009; Wu, 2013). However, the effect of avidity on the intracellular interactome and its spatio-temporal regulation has been less well-described compared to the extracellular examples above. In fact, the functional consequence of multivalency in intracellular signaling complexes has been largely neglected and few reports directly addresses the existence or origin of the avidity. As for extracellular interactions, multivalency, hindered diffusion and reduced dimensionality maintains high effective concentrations and increases residence time of the involved complexes. In particular, large cellular structures such as the plasma and intracellular membranes, DNA and the cytoskeleton provide perfect frameworks for tethering binding sites which can be coordinated by bi- or multivalent binders. Moreover, intracellular multivalent interactions are to a larger extent heterovalent, which greatly enhance the specificity, and change the retention time at sites where two ligands are clustered compared to when they are not. In membranes, also the local concentration of potential binding sites and thereby also the avidity and retention time is largely affected by diffusion rates of the ligands within the membrane.

From a biological perspective, multivalency and resulting avidity can dramatically alter cellular function. As discussed above, multivalent complexes can have very long retention and equilibration times which can control both duration and strength of a given cellular process. Gene transcription and transcriptional regulation are good examples of this as DNA contains thousands of different specific and repeatable binding motifs for transcription and transcriptional regulation of genes. Although binding motifs can be more or less exposed as a consequence of DNA compaction/condensation, the binding motifs are fixed relative to each other and are often located close together yielding very high local concentrations (0.1–1 M). An example is the *Escherichia coli* RNA polymerase (RNAP) which binds (at least) three sites designated −35, −10, and upstream element, UP. The RNAP binds different promotors with avidities spanning three orders of magnitude depending on the primary base sequence and the space between the sites (Dayton et al., 1984; Liu et al., 2003). A recent study by *Einav and Phillips* analyzed gene expression of over 10,000 promoters spanning all possible combinations of a set of regulatory elements (Urtecho et al., 2019). They showed that discrepancies between experimental and predicted expression levels could be explained by applying a multivalent model describing how the RNAP can buffer mutations but also that too tight binding can inhibit gene transcription (Einav and Phillips, 2019). Moreover it was also shown that the local concentration of RNAP leads to different mechanisms of diffusion of RNAP which has a dominating effect on how specific target sites are located (Wang et al., 2013).

Another example more classical example is the Retinoid X Receptor transcription factor (RXR). RXR belongs to the family of nuclear hormone receptors (NHR) which are ligand-activated transcription factors that regulate gene expression by interacting with specific repetitive DNA sequences (hormone receptor elements; HREs) upstream of their target genes. NHRs bind as dimers with each monomer recognizing a six base pair sequence of DNA. The intrinsic affinity of the RXR monomer for DNA is roughly 1 μM whereas the RXR homodimers vary from 10 to 160 nM for different nucleotide sequences (Osz et al., 2015). Also here, the transcription level scales non-linearly with the avidity and it has also been found that RXR tetramers may in some cases be required for transcription to take place (Chen and Privalsky, 1995).

Similar to DNA, cellular membranes are capable of tethering and clustering ligands for multivalent binders. Like protein-nucleic acid interactions above, interactions with membranes themselves (protein-lipid interaction) or membrane embedded ligands (protein-protein interaction) are sensitive to compositional and structural changes. Additionally, membranes are fluid and ligands diffuse within the membrane with rates much slower than in solution and only in two dimensions (Javanainen et al., 2017).

A vast variety of multivalent molecules interact with membranes or membrane tethered ligands. Scaffolding proteins are multidomain molecular hubs important for many cellular processes including cell-cell signaling, intracellular signaling and assembly line processes. Cellular localization of receptors, cellular adhesion molecules and their intracellular association with enzymes or downstream signaling molecules are largely controlled by specific interactions with scaffolding protein domains (Good et al., 2011). Scaffolding protein domains are usually protein-protein interacting modules but are also in some cases capable of specifically binding lipids or even cellular structures (Erlendsson and Madsen, 2015; Herlo et al., 2018). Canonical scaffolding domains include; SH2/3, PX, PH, and PDZ domains. Scaffolding proteins such as GRIP14 and MUPP115 contain almost exclusively PDZ domains (7 and 13, respectively) arranged like beads on a string, whereas PSD-95, CASK, and MAGI contains at least one PDZ domain, and one or more SH3/WW domains. The N-BAR family of scaffolding proteins including PICK1 and endophilin also comprises a coiled-coil dimerization/oligomerization domain.

One study used TIRF microscopy to probe the near membrane dwell-time (retention time) of the scaffolding protein Grb2, containing two SH3 domains and one SH2 domain. The SH2 domain binds to phosphotyrosines whereas the SH3 domains binds proline rich regions. In good agreement with the rebinding model they found that Grb2 resides significantly longer than predicted from the intrinsic chemical dissociation rate constant between phosphotyrosine and the Grb2 SH2 domain. It was also found that the dwell time is positively correlated with the local density of receptor tyrosine kinase phosphorylation (Oh et al., 2012).

For PICK1 and PSD95 we have recently used a cell membrane sheet assay to investigate the ligand binding of PICK1 and PSD-95 on the inner leaflet of the plasma membrane (Erlendsson et al., 2019). These experiments showed that PICK1 and PSD-95 bind their partner proteins with avidities up to two orders of magnitude better than the intrinsic affinities previously observed using other binding assays. We also found that changing the intrinsic affinity from 1 to 10 μM, resulted in a complete switch between a multivalent and monovalent interaction mode at physiological protein concentrations, and that only one of these modes resulted in recycling of ligand receptors. Combining these experiments with kinetic simulations similar to those presented above, we were able to demonstrate clear biphasic behavior of this system and also determine the microscopic reaction rates governing the avidity. In good agreement with theory we showed that the retention time and equilibration time becomes extremely long (t_1/2_, ~ 375 min, *k*_on,av_ ~ 25 min at a concentration of 5 times *K*_av_) (Erlendsson et al., 2019). From this insight, a high avidity bivalent inhibitor of PICK has recently been developed that alleviate neuropathic pain in rats (Christensen et al., 2020).

## Flexible Multivalent Systems

The examples above relate to multidomain proteins binding to other multidomain or tethered ligands with highly specific binding motifs, however, recently the principles of avidity and multivalency has also been expanded to include interactions involving fully flexible systems such as intrinsically disordered proteins (IDPs), and systems with less specific binding sequences e.g., phosphorylation sites or short linear motifs (SLiMs) (Mittag et al., 2008; Tompa, 2014; Bugge et al., 2020). SLiMs do not have extensive interaction interfaces to induce high enthalpy so instead SLiM-containing IDPs often utilize multiple motifs to participate in multivalent interactions (Hayama et al., 2018).

One example is the bivalent 14-3-3 protein scaffold, which interacts with a wide range of signaling proteins via phosphorylated serine residues. Ligands include the Cystic Fibrosis Transmembrane conductance Regulator (CFTR) and Leucine-Rich Repeat Kinase 2 (LRRK2) featuring nine and six 14-3-3 binding motifs, respectively. The avidity gain spans 3 orders of magnitude for CTFR (1.1 × 10^−3^-2.9 × 10^−6^ M) but almost 6 orders of magnitude for LRRK2 (8.6 × 10^−4^ to 2.8 × 10^−10^ M). This difference is strongly correlated with the effective concentration of the binding sites, which ranges from 1.2 to 110 mM, and the authors find a strong but different enthalpy–entropy correlation for the two systems (Stevers et al., 2018).

Another example relates to transport across the nuclear pore complex (NPC). The central channel of the NPC is lined by intrinsically disordered regions of Phe–Gly nucleoporins (FG Nups) which form a selective permeable barrier for macromolecular transport. FG Nups typically contain 5–50 Phe-Gly motifs separated by spacer residues. In spite of many FG repeats the avidity is not as dramatic as for the other multivalent systems presented above. The intrinsic affinity between the nuclear transport factor 2 (NTF2) and one FG motif is ~3 mM which increases to ~0.5 mM for four FG motifs, but does not increase any further if additional FG motifs are introduced. Similar to the 14-3-3 proteins, the enthalpy–entropy balance prevents strong avidity which enables rapid and reversible interactions necessary for transport across the NPC. However, the enthalpy–entropy balance can be compensated by a high local concentration of FG motifs which permits higher frequency of contacts and thereby longer residence time (Hayama et al., 2018).

## Linkers and Local Concentrations

In the thermodynamic model, the avidity contribution from interactions between the linker(s) of the ligand and the receptor are given by its enthalpy, Δ${\text{H}}_{\text{linker},}^{0}$ and conformational entropy, Δ${\text{S}}_{\text{linker}}^{0}$ (Kitov and Bundle, 2003; Krishnamurthy et al., 2006; Kane, 2010). In the kinetic models, the length of the linker(s) dictates the minimum local concentration (the binding sites might be closer in space than the reach of the linker) and the conformational enthalpy and entropy is embedded in the composite penalty parameter, *f*. For most flexible linkers the enthalpic interactions between the linker and the protein binding is neglectable and therefore the linker contribution to the overall avidity is almost entirely entropic. For fully flexible linkers the change in conformational entropy is low (Krishnamurthy et al., 2007), but may increase significantly if the valency is high (Hayama et al., 2018), or if the composition or the structure of the peptide or nucleic acid changes (van Leeuwen et al., 1997; Einav et al., 2019; Sørensen and Kjaergaard, 2019). In most cases multivalent ligands with flexible linkers longer than the distance between the binding sites will be less effective in binding as the distance increases. For this reason, using local concentrations calculated based on the distance between the binding sites gives a good estimate of the theoretical avidity of the system (Krishnamurthy et al., 2007).

However, when calculating the local concentration, we make the assumption that all distances within the reach of the multivalent molecule are equally well-populated. This is essentially not the case. In a bivalent system, shortening or lengthening the distance between available binding sites changes the effective local concentration that modulates the secondary binding event. Shorter distances translate into higher effective local concentrations and *vice versa*. In situations where binding is not limited by the availability of binding sites, determining which local concentration zones are mostly populated depends ultimately on the flexibility and chemical composition of the linker itself. Multivalent systems can range from disordered, where all configurations are equally favored, to rigid where only certain configurations are allowed.

To more explicitly take into account the steric effects of the linker when calculating the local concentration, Errington et al. recently devised a more sophisticated model taking into account the uneven linker end-to-end distribution and the probability of binding between the ligand and receptor (Errington et al., 2019). Here the effective concentration (local concentration) of all receptor/ligand pairs can be calculated by using the probability density functions (PDF). PDFs and therefore specific local concentrations can be generated for each individual states/configuration within the multivalent binding network. This approach was able to accurately predict experimental avidities and model microstates with different abundances and lifetimes.

## Emerging Concepts and Future Perspectives

In addition to the effects we have described in this review, multivalency may also lead to a series of other properties not observed for monovalent protein-ligand complexes. Designing multivalent drugs have huge potential and is already widely applied (Mourez et al., 2001; Kim et al., 2008; Bach et al., 2012; Dubacheva et al., 2015; Karlsson et al., 2015; Maric et al., 2015; Einav et al., 2019; Christensen et al., 2020; Ortega et al., 2020). Within the past 5 years it has become increasingly clear how multivalent interactions of particularly RNA and IDP's drive liquid-liquid phase separations (Cohan and Pappu, 2020; Conicella et al., 2020; Guillén-Boixet et al., 2020; Martin et al., 2020; Ryan et al., 2020). Multivalent interactions can also give rise to superselectivity of synthetic ligands where several weak binding sites are linked together (Martinez-Veracoechea and Frenkel, 2011; Dubacheva et al., 2015). These systems shift between bound and unbound states over a very narrow concentration range and have great potential for use as detectors in nanodevices (Wang et al., 2010, 2020; Foster et al., 2017; Curk et al., 2020) much similar to what can be achieved by super cooperative aptamers and proteins (Ortega et al., 2020).

In this review, we have shown examples of biological systems inside cells where the high avidity and long residence time typical for multivalent interactions are important for the biological function. Intracellular interactions are particularly challenging to analyse as it poses a great technical challenge to titrate an intracellular receptor with ligand without disturbing the native environment. This is important e.g., in the postsynaptic density where many supramolecular complexes form that depend on membrane embedded receptors and the right composition and topology of the membrane. Developing methodology for extracting accurate thermodynamic data in native-like environments will be a great step toward a more detailed understanding of how the activity of such complexes are regulated. Future experiments making use of molecular dynamics simulations in appropriate force fields could also potentially allow for even more accurate predictions of local concentrations and regulation by multivalency.

## Author Contributions

All authors listed have made a substantial, direct and intellectual contribution to the work, and approved it for publication.

## Conflict of Interest

The authors declare that the research was conducted in the absence of any commercial or financial relationships that could be construed as a potential conflict of interest.
